# The 2017–2018 influenza season in Bucharest, Romania: epidemiology and characteristics of hospital admissions for influenza-like illness

**DOI:** 10.1186/s12879-019-4613-z

**Published:** 2019-11-12

**Authors:** Anca Drăgănescu, Oana Săndulescu, Dragoș Florea, Ovidiu Vlaicu, Anca Streinu-Cercel, Dan Oțelea, Monica Luminița Luminos, Victoria Aramă, Sorin Abrudan, Adrian Streinu-Cercel, Daniela Pițigoi

**Affiliations:** 1 Children X Department, National Institute for Infectious Diseases ‘Prof. Dr. Matei Balş’, Bucharest, Romania; 2 Adults II Department, National Institute for Infectious Diseases ‘Prof. Dr. Matei Balş’, Bucharest, Romania; 3 Department of Infectious Diseases I, Faculty of Medicine, Carol Davila University of Medicine and Pharmacy, Bucharest, Romania; 4 Molecular Diagnosis Laboratory, National Institute for Infectious Diseases ‘Prof. Dr. Matei Balş’, Bucharest, Romania; 5 Department of Microbiology I, Faculty of Medicine, Carol Davila University of Medicine and Pharmacy, Bucharest, Romania; 6 Department of Infectious Diseases, Faculty of Dental Medicine, Carol Davila University of Medicine and Pharmacy, Bucharest, Romania; 7 Adults III Department, National Institute for Infectious Diseases ‘Prof. Dr. Matei Balş’, Bucharest, Romania; 8 Medical CEE, Sanofi Pasteur, Bucharest, Romania; 9 Compartment for Surveillance and Prevention of Healthcare-associated Infections, National Institute for Infectious Diseases ‘Prof. Dr. Matei Balş’, Bucharest, Romania; 10 Department of Epidemiology, Faculty of Medicine, Carol Davila University of Medicine and Pharmacy, Bucharest, Romania

**Keywords:** Children, Comorbidity, Epidemiology, Hospitalization, Influenza, Older adults, Romania

## Abstract

**Background:**

Seasonal influenza causes a considerable burden to healthcare services every year. To better measure the impact of severe influenza cases in Romania, we analyzed active surveillance data collected during the 2017–2018 season from patients admitted for influenza-like illness (ILI) at a tertiary care hospital in Bucharest.

**Methods:**

Patients admitted for acute ILI were included if they were resident in the Bucharest-Ilfov region, had been hospitalized for at least 24 h, and had onset of symptoms within 7 days before admission. Patient demographics, healthcare use, vaccination status, and outcome data were collected by questionnaire or by searching clinical records. Respiratory swabs were also obtained from each patient to confirm influenza A (A/H1 and A/H3 subtypes) or influenza B (Yamagata and Victoria lineages) infection by real-time reverse-transcription polymerase chain reaction assay.

**Results:**

The study included 502 patients, many (45.2%) of whom were aged < 5 years. Overall, 108 patients (21.5%) had one or more comorbidities. Seventeen adults aged 18–64 years (3.4%) had been vaccinated against influenza. Patients were hospitalized for a median of 5 days and most (90.4%) were prescribed antiviral treatment. More than one-half of the patients (*n* = 259, 51.6%) were positive for influenza. Most influenza cases were caused by B viruses (172/259, 66.4%), which were mostly of the B/Yamagata lineage (85 of 94 characterized, 90.4%). Most of the subtyped A viruses were A/H1 (59/74, 79.7%). A/H1 viruses were frequently detected in influenza-positive admissions throughout the 2017–2018 season, whereas the predominant B/Yamagata viruses were detected around the middle of the season, with a peak in cases at week 7 of 2018. Eleven patients were admitted to an intensive care unit; of these, one patient with confirmed B/Yamagata infection died.

**Conclusions:**

These results show that seasonal influenza results in considerable hospitalization in Bucharest-Ilfov, Romania and suggest vaccine coverage should be extended, especially to the youngest age groups. The data from this study should help inform and optimize national influenza healthcare policies.

## Background

Influenza epidemics are a frequent cause of severe illness and death, particularly in older adults, young children, and persons with chronic health conditions [1, 2]. Seasonal influenza is caused by influenza type A and type B viruses, which co-circulate around the world and continually evolve to form new strains [2]. Yearly vaccination against the predominant circulating A and B viruses is the most effective measure for protecting against seasonal influenza and its complications [1]. In line with World Health Organization (WHO) recommendations, most national health authorities recommend influenza vaccination before the start of each season for those at greatest risk of influenza complications [1, 3].

Because the burden of disease can vary between influenza seasons and countries, detailed epidemiologic data are needed for each season to guide national vaccination policies and evaluate how effective they are. Epidemiologic data may be particularly important for evidence-based policy decisions in low- and middle-income countries with limited healthcare resources for allocation [4]. Although the disease burden from seasonal influenza is frequently characterized in Western Europe, far less epidemiologic data has been generated for populations in Eastern European and Central European countries [4].

Romania is one such country for which the seasonal burden of influenza is incompletely defined. Between 2011–2012 and 2015–2016, Romania was estimated to have an overall annual incidence of 68–318 cases of influenza-like illness (ILI) requiring medical attention per 100,000 population, with 27%–56% of these cases confirmed as influenza [5]. Over the same time period, between 317 and 625 individuals were hospitalized in each season for influenza-associated severe acute respiratory infection (SARI) [5]. The rate of mortality among these patients with confirmed influenza ranged from 1.4% in 2011–2012 to as high as 39.8% in 2015–2016.

Influenza vaccination is provided by health authorities in Romania to persons aged 65 years and older, patients with comorbidities, pregnant women, institutionalized persons, and social and healthcare workers [6, 7]. Despite this, Romania has an influenza vaccination coverage rate well below the WHO’s 75% target for key risk groups [6, 8]. Recent national estimates suggest vaccination among Romanians aged 65 years and older was 16.3% in 2017–2018 [9] and only 8.2% in 2016–2017 [10].

To better measure the impact of severe influenza cases in Romania, we analyzed active surveillance data collected during the 2017–2018 season from patients admitted for ILI at a tertiary care hospital in Bucharest. Here, we report the demographics, symptoms, healthcare use, and outcomes for these patients and the distribution of influenza virus strains detected.

## Methods

### Study design

This was an observational, hospital-based, active surveillance study of patients presenting with ILI from the Bucharest-Ilfov region. The study was conducted at the National Institute for Infectious Diseases ‘Prof. Dr. Matei Balș’, Bucharest, Romania during the 2017–2018 influenza season. The study site is one of two infectious disease hospitals covering the Bucharest-Ilfov region, and the main referral center for the diagnosis and management of patients with influenza throughout the country. Inclusion of ILI patients followed the Global Influenza Hospital Surveillance Network core protocol [11], in which for patients ≥5 years the ILI case definition is based on that used by the European Centre for Disease Prevention and Control [12]. Briefly, the study included patients admitted for acute ILI who had been hospitalized for at least 24 h, with the onset of symptoms within 7 days prior to admission. In patients aged ≥5 years, systemic symptoms had to include at least one of the following: fever (or feeling hot), headache, myalgia, or malaise, and at least one of the following respiratory symptoms: cough, sore throat, or shortness of breath. Patients aged < 5 years were included if their admission diagnoses were among the International Classification of Diseases 10th revision (ICD-10) codes referenced by the Global Influenza Hospital Surveillance Network core protocol [11, 13], with the onset of symptoms within 7 days prior to admission. ILI symptoms were not collected from patients aged < 5 years. Potential subjects were excluded if they did not live in the Bucharest-Ilfov area, were institutionalized, or had been discharged from a hospital within 30 days of the current admission.

### Study conduct

For each patient, a common standardized questionnaire was completed by face-to-face interview and follow-up information was completed by searching clinical records. Collected information included age, sex, number and type of comorbidities, smoking habits, admission and discharge dates and diagnoses, ILI symptoms at admission, previous admissions to a hospital in the last 12 months, number of visits to a general practitioner in the last 3 months, treatment received, and outcome of infection. Influenza vaccination status for the current and previous two seasons was also collected. Two respiratory swabs were obtained from each patient: for patients aged < 14 years, nasopharyngeal and nasal swabs were collected; for patients aged ≥14 years, nasopharyngeal and pharyngeal swabs were obtained. Real-time reverse-transcription polymerase chain reaction assays were used to detect and differentiate influenza A (A/H1 and A/H3 subtypes), influenza B (Yamagata and Victoria lineages), and respiratory syncytial virus (RSV) from the swabs, as previously described [7].

### Statistical analysis

All data were analyzed using Excel (Microsoft, Redmond, WA, US). Missing data were not replaced, and only descriptive statistics were calculated. Continuous variables with non-parametric distribution are reported using the median and interquartile range (IQR). Categorical variables are reported as frequencies and percentages.

## Results

### Patient characteristics

**Fig. 1:**
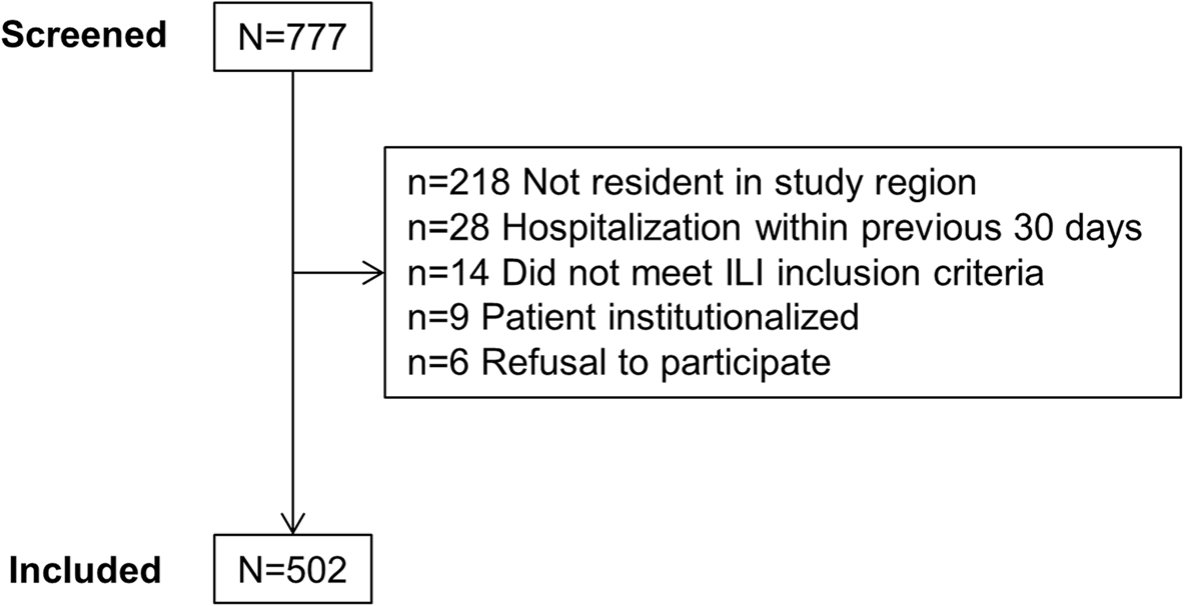
Patient flow chart

Patients with ILI were screened between December 11, 2017 and April 30, 2018. Out of 777 patients screened, 502 met the inclusion criteria and were tested for influenza (Fig. 1). More than one-quarter of screened patients (218/777, 28.1%) were excluded because they did not live in the Bucharest-Ilfov region.

Patients presented to the hospital a median of 2 days (IQR, 1–4) after symptom onset. A large proportion of admissions were aged less than 5 years (*n* = 227, 45.2%) (Table 1). The proportions of male and female patients were similar in most age groups. Overall, 34.4% of patients had been hospitalized in the previous 12 months and most (51.5%) had received outpatient consultations within 3 months before admission.

**Table 1:** Characteristics of patients by age group and overall

Characteristic	< 1 y *N* = 39	1–2 y *N* = 111	3–4 y *N* = 77	5–13 y *N* = 103	14–17 y *N* = 7	18–64 y *N* = 135	≥65 y *N* = 30	Overall *N* = 502
Age (y), median [IQR]	0.7 [0.4–0.9]	1 [1–2]	4 [3–4]	7 [6–9]	15 [15–16.5]	45 [35–55]	76 [68.3–80.8]	5.5 [2–36]
Sex, *n* (%)								
Male	20 (51.3)	60 (54.1)	41 (53.2)	57 (55.3)	5 (71.4)	46 (33.8)	15 (50.0)	244 (48.6)
Female	19 (48.7)	51 (45.9)	36 (46.8)	46 (44.7)	2 (28.6)	89 (65.9)	15 (50.0)	258 (51.4)
Smoking status, *n* (%)^a^								
Never smoked	25 (64.1)	27 (24.3)	44 (57.1)	63 (61.2)	7 (100.0)	61 (45.2)	10 (33.3)	282 (56.2)
Past smoker	4 (10.3)	12 (10.8)	9 (11.7)	8 (7.8)	0 (0.0)	32 (23.7)	15 (50.0)	80 (15.9)
Current smoker	10 (25.6)	72 (64.9)	24 (31.2)	32 (31.1)	0 (0.0)	42 (31.1)	5 (16.7)	140 (27.9)
Comorbidities, *n* (%)								
Any	1 (2.6)	6 (5.4)	2 (2.6)	8 (7.8)	2 (28.6)	64 (47.4)	25 (83.3)	108 (21.5)
Cardiovascular disease	0 (0.0)	1 (0.9)	0 (0.0)	0 (0.0)	0 (0.0)	30 (22.2)	23 (76.7)	54 (10.8)
COPD	0 (0.0)	0 (0.0)	0 (0.0)	0 (0.0)	0 (0.0)	6 (4.4)	4 (13.3)	10 (2.0)
Asthma	0 (0.0)	1 (0.9)	1 (1.3)	4 (3.9)	0 (0.0)	3 (2.2)	1 (3.3)	10 (2.0)
Diabetes	0 (0.0)	0 (0.0)	0 (0.0)	1 (1.0)	1 (14.3)	11 (8.1)	6 (20.0)	19 (3.8)
Immunodeficiency	1 (2.6)	3 (2.7)	1 (1.3)	1 (1.0)	1 (14.3)	11 (8.1)	0 (0.0)	18 (3.6)
Renal impairment	1 (2.6)	0 (0.0)	0 (0.0)	1 (1.0)	0 (0.0)	5 (3.7)	2 (6.7)	9 (1.8)
Autoimmune disease	0 (0.0)	0 (0.0)	0 (0.0)	0 (0.0)	0 (0.0)	10 (7.4)	2 (6.7)	12 (2.4)
Neuromuscular disease	0 (0.0)	1 (0.9)	0 (0.0)	0 (0.0)	0 (0.0)	6 (4.4)	7 (23.3)	14 (2.8)
Cirrhosis/liver disease	0 (0.0)	0 (0.0)	0 (0.0)	0 (0.0)	0 (0.0)	21 (15.6)	1 (3.3)	22 (4.4)
Neoplasm	0 (0.0)	0 (0.0)	0 (0.0)	1 (1.0)	0 (0.0)	3 (2.2)	2 (6.7)	6 (1.2)
Pregnant, *n* (%)	–	–	–	–	0 (0.0)	7 (5.2)	–	7 (1.4)
ILI symptoms, *n* (%)								
Fever	–	–	–	101 (98.1)	7 (100.0)	109 (80.7)	25 (83.3)	242 (88.4)^b^
Malaise	–	–	–	90 (87.4)	7 (100.0)	128 (94.8)	27 (90.0)	252 (91.6)^b^
Headache	–	–	–	70 (68.0)	6 (85.7)	109 (80.7)	17 (56.7)	202 (73.5)^b^
Myalgia	–	–	–	39 (37.9)	5 (71.4)	99 (73.3)	19 (63.3)	162 (58.9)^b^
Cough	–	–	–	92 (89.3)	6 (85.7)	111 (82.2)	27 (90.0)	236 (85.8)^b^
Sore throat	–	–	–	77 (74.8)	7 (100.0)	77 (57.0)	11 (36.7)	172 (62.5)^b^
Shortness of breath	–	–	–	15 (14.6)	4 (57.1)	65 (48.1)	21 (70.0)	105 (38.2)^b^
Tachypnea^c^	2 (5.1)	11 (9.9)	15 (19.5)	–	–	–	–	28 (12.3)^d^
Hospitalizations in previous 12 mo, *n* (%)								
1	8 (20.5)	30 (27.0)	22 (28.6)	19 (18.4)	1 (14.3)	14 (10.4)^e^	10 (33.3)	104 (20.8)^f^
≥ 2	8 (20.5)	19 (17.1)	14 (18.2)	11 (10.7)	1 (14.3)	10 (7.5)^e^	5 (16.7)	68 (13.6)^f^
Outpatient consultations in previous 3 mo, *n* (%)								
1	11 (29.0)^g^	26 (23.4)	15 (19.7)^h^	17 (16.5)	2 (28.6)	22 (16.4)^e^	8 (26.7)	101 (20.2)^i^
≥ 2	11 (29.0)^g^	50 (45.0)	34 (44.7)^h^	41 (39.8)	1 (14.3)	14 (10.4)^e^	5 (16.7)	156 (31.3)^i^
Vaccinated against influenza in 2017–2018, *n* (%)	0 (0.0)^j^	0 (0.0)	0 (0.0)	0 (0.0)	0 (0.0)	17 (12.6)	0 (0.0)	17 (3.4)
≥ 14 days before ILI onset^k^	0 (0.0)	0 (0.0)	0 (0.0)	0 (0.0)	0 (0.0)	10 (7.4)	0 (0.0)	10 (2.0)
Patients with comorbidities^l^	0 (0.0)	0 (0.0)	0 (0.0)	0 (0.0)	0 (0.0)	8 (12.5)	0 (0.0)	8 (7.4)
Vaccinated in previous seasons, *n* (%)								
2016–2017	0 (0.0)	0 (0.0)	0 (0.0)	0 (0.0)	0 (0.0)	14 (10.4)	1 (3.3)	15 (3.0)
2015–2016	0 (0.0)	0 (0.0)	0 (0.0)	0 (0.0)	0 (0.0)	17 (12.6)	2 (6.7)	19 (3.8)
Duration of hospitalization (days), median [IQR]	5 [4–6.75]^g^	5 [3–6.5]	5 [4–6]	5 [4–7]^m^	5 [4.5–8.5]	5 [3–7]	7 [6–9.5]	5 [3–7]^i^
Antiviral treatment prescribed, *n* (%)	38 (97.4)	108 (97.3)	76 (98.7)	99 (96.1)	5 (71.4)	106 (78.5)	22 (73.3)	454 (90.4)
Intensive care received, *n* (%)								
ICU admission	0 (0.0)	4 (3.6)	1 (1.3)	2 (1.9)	0 (0.0)	4 (3.0)	0 (0.0)	11 (2.2)
Mechanical ventilation	0 (0.0)	0 (0.0)	0 (0.0)	0 (0.0)	0 (0.0)	1 (0.7)	0 (0.0)	1 (0.2)
Death while hospitalized, *n* (%)	0 (0.0)	0 (0.0)	0 (0.0)	0 (0.0)	0 (0.0)	1 (0.7)	0 (0.0)	1 (0.2)

*Abbreviations*: *COPD* Chronic obstructive pulmonary disease, *ICU* Intensive care unit, *ILI* Influenza-like illness, *IQR* Interquartile range

^a^ For patients aged 0–13 years of age, smoking habits refer to parents or tutors

^b^ Percentage of patients aged ≥5 years (*N* = 275)

^c^ Recorded only for patients aged 0–4 years. For patients aged 0–11 months, defined as > 50 breaths per minute; for patients 12–60 months, defined as > 40 breaths per minute

^d^ Percentage of patients aged 0–4 years (N = 227)

^e^ *N* = 134

^f^ *N* = 501

^g^ *N* = 38

^h^ *N* = 76

^i^ *N* = 499

^j^ Influenza vaccination was contraindicated for 17 patients because they were aged < 6 months

^k^ Percentage of patients in each age group

^l^ Percentage of patients with comorbidities

^m^ *N* = 101

Overall, 108 patients (21.5%) had one or more comorbidities. Most patients aged over 65 years had comorbidities (25/30; 83.3%), the most common being cardiovascular disease (in 76.7%). Comorbidities were less common among patients younger than 18 years. Overall, around one-quarter of the patients (or the parents of patients younger than 14 years) were current smokers. Seven women were pregnant at admission.

The most common symptoms of ILI among patients aged 5 years and older were malaise (91.6%), fever (88.4%), cough (85.8%), and headache (73.5%). Tachypnea was reported in 12.3% of admissions aged 0–4 years.

Seventeen adults aged 18–64 years (3.4% of all patients) had been vaccinated against influenza for the present 2017–2018 season, of whom 10 had received their vaccine 14 days or longer before ILI onset. Eight of the 108 patients (7.4%) with a comorbidity, and none of the 30 patients aged 65 years and older, had been vaccinated for the present season. Similarly, only two adults aged 65 years and older reported having received an influenza vaccine in either of the two preceding influenza seasons. None of the hospitalized pregnant women had been vaccinated.

### Healthcare use and outcome

Patients were hospitalized for a median of 5 days (IQR, 3–7 days) (Table 1). Patients aged 65 years and older were hospitalized for longer (median, 7 days [IQR, 6–9.5 days]). Most patients (90.4%) were prescribed antiviral treatment during their hospitalization, particularly those aged 0–13 years (> 96%). In cases where antiviral treatment was administered, it was started immediately after swab collection.

Eleven patients were admitted to an intensive care unit (ICU), one of whom also required mechanical ventilation. One of the patients died after ICU admission; he had confirmed influenza B/Yamagata infection, more than one chronic underlying health condition, and had not been vaccinated against influenza for the current season.

### Characteristics of laboratory-confirmed influenza cases

Respiratory swabs were obtained from all included patients for influenza virus detection. More than half of the patients (*n* = 259, 51.6%) were positive for influenza (Table 2). RSV was confirmed in 48 patients, most of whom (*n* = 39, 81.3%) were 0–4 years of age. Six patients were co-infected with RSV and influenza B. Demographics, symptoms, healthcare use, and outcomes were broadly similar between influenza-positive and -negative admissions, except that influenza-positive patients reported cough more frequently than influenza-negative patients (92.8% vs. 77.2%) and more were admitted to an ICU (eight influenza-positive patients vs. three influenza-negative patients [3.1% vs. 1.2%]).

**Table 2:** RT-PCR results by age group and overall

Category	< 1 y *N* = 39	1–2 y *N* = 111	3–4 y *N* = 77	5–13 y *N* = 103	14–17 y *N* = 7	18–64 y *N* = 135	≥65 y *N* = 30	Overall *N* = 502
Laboratory-confirmed influenza	19 (48.7)	46 (41.4)	42 (54.5)	66 (64.1)	5 (71.4)	66 (48.9)	15 (50.0)	259 (51.6)
Influenza A	7 (17.9)	18 (16.2)	28 (36.4)	15 (14.6)	0 (0.0)	16 (11.9)	3 (10.0)	87 (17.3)
A/H1	6 (15.4)	15 (13.5)	22 (28.6)	10 (9.7)	0 (0.0)	5 (3.7)	1 (3.3)	59 (11.8)
A/H3	1 (2.6)	1 (0.9)	4 (5.2)	3 (2.9)	0 (0.0)	6 (4.4)	0 (0.0)	15 (3.0)
A/not subtyped	0 (0.0)	2 (1.8)	2 (2.6)	2 (1.9)	0 (0.0)	5 (3.7)	2 (6.7)	13 (2.6)
Influenza B	12 (30.8)	28 (25.2)	14 (18.2)	51 (49.5)	5 (71.4)	50 (37.0)	12 (40.0)	172 (34.3)
B/Yamagata	6 (15.4)	14 (12.6)	8 (10.4)	29 (28.2)	2 (28.6)	22 (16.3)	4 (13.3)	85 (16.9)
B/Victoria	0 (0.0)	2 (1.8)	0 (0.0)	6 (5.8)	0 (0.0)	1 (0.7)	0 (0.0)	9 (1.8)
B/not characterized	6 (15.4)	12 (10.8)	6 (7.8)	16 (15.5)	3 (42.9)	27 (20.0)	8 (26.7)	78 (15.5)
Laboratory-confirmed RSV	9 (23.1)	19 (17.1)	11 (14.3)	5 (4.9)	0 (0.0)	2 (1.5)	2 (6.7)	48 (9.6)^a^

*Abbreviations*: *RSV* Respiratory syncytial virus, *RT-PCR* Real-time reverse-transcription polymerase chain reaction

^a^ Six patients had RSV and influenza B coinfection

Two-thirds of laboratory-confirmed influenza cases were caused by B viruses (172/259, 66.4%), of which almost all those characterized were of the B/Yamagata lineage (85/94, 90.4%). Most of the subtyped A viruses were A/H1 (59/74, 79.7%). With the exception of 3–4-year-olds, where a higher proportion of influenza A cases was observed, the proportions of influenza viruses and strains detected did not differ across the age groups.

Five patients with laboratory-confirmed influenza had received the 2017–2018 influenza vaccine 14 days or longer before symptom onset; all five had received a trivalent inactivated influenza vaccine (Additional file 1: Table S1). B/Yamagata, the B lineage not included in the vaccine, was responsible for four of these cases and a B virus of undefined lineage for one.

**Fig. 2:**
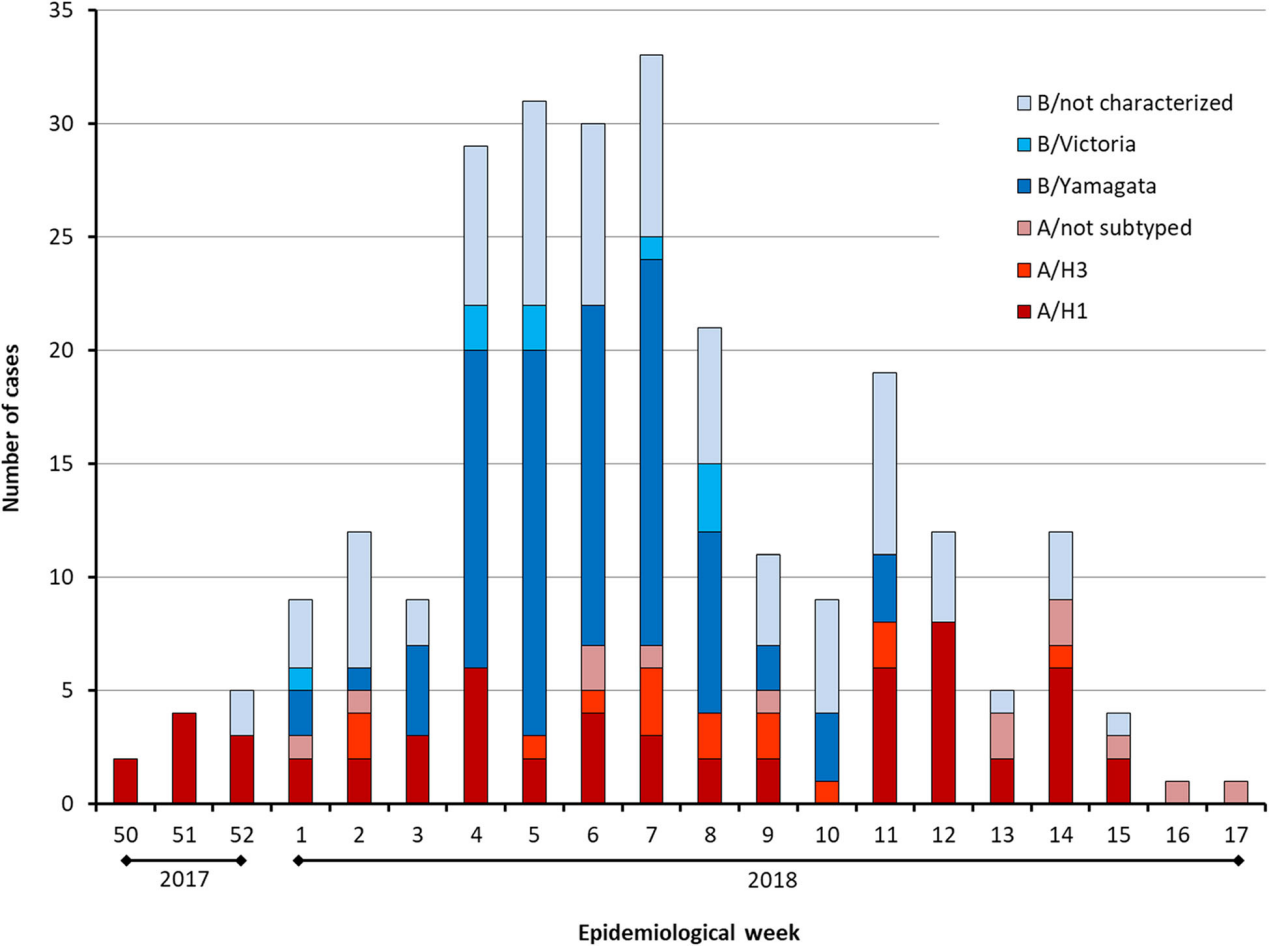
Admissions with influenza by epidemiologic week and virus type, subtype, or lineage

The first cases of confirmed influenza were detected in week 50 of 2017 (Fig. 2). Influenza A/H1 accounted for most cases during the first few weeks of the season. From the start of 2018, the proportion of cases involving influenza B began to increase. Week 7 of 2018 had the highest number of cases, most of which were associated with B/Yamagata viruses. After this peak, the proportion of cases caused by influenza B decreased, whereas cases caused by influenza A (notably A/H1) continued through 2018 until the end of the season at week 17.

## Discussion

This study showed that ILI, particularly ILI due to influenza, results in a considerable number of hospital admissions in Romania’s Bucharest-Ilfov region. Healthcare use was substantial; patients were hospitalized for a median of 5 days, almost all patients received antiviral treatment during their hospitalization, and 11 patients (2.2% overall) required admission to an ICU.

Almost one-half of the patients were less than 5 years of age, and more than one-fifth of all patients had comorbidities. Influenza hospitalizations of young, otherwise healthy children have been frequently reported in previous seasons at the same hospital [7] and at other public healthcare facilities in Romania [5]. Young children have a higher risk of developing severe influenza illness as well as other complications (e.g., bacterial superinfections [14]), which emphasizes the importance of vaccination for this age group [1]. Individuals with chronic illnesses also account for a large proportion of influenza hospitalizations in Romania. In the 2011–2012 to 2015–2016 seasons, 51%–66% of patients hospitalized for influenza-positive SARI in Romania suffered from underlying medical conditions [5]. Surveillance data from the 2009–2010 and 2010–2011 seasons also found a greater risk of death among Romanian patients hospitalized with influenza A/H1N1 infection if they had underlying comorbidities [15]. Most patients aged over 65 years in our study had underlying medical conditions, most frequently cardiovascular disease, neuromuscular disease, or diabetes. A recent study from Romania has shown that elderly patients with diabetes require hospitalization for SARI at a higher rate compared to the general elderly population [16].

In the current study, most cases of influenza in hospitalized patients were due to influenza B/Yamagata viruses, although A/H1 viruses were also frequently detected. This pattern matches national data reported in Romania [17] and the influenza virus circulation patterns across Europe in 2017–2018, which were characterized by longer and higher levels of virus circulation than previous influenza seasons [18]. Influenza virus circulation progressed in a marked west-to-east direction in 2017–2018, with countries in eastern Europe advised early during the season to prepare for possible cases of severe disease and heavy impacts on healthcare services [19]. Consistent with this, we found the 2017–2018 season started relatively late in the Bucharest-Ilfov region, first with A/H1 type influenza viruses predominant in December 2017 followed by a predominance of B/Yamagata virus at the season’s peak in mid-February 2018. A similar biphasic predominance was observed during the previous 2016–2017 season among hospitalized patients from the same region, although by contrast, only B/Victoria-lineage and A/H3 viruses were identified [7]. Also, unlike previous seasons at our hospital [7, 14], influenza A viruses were detected in ILI admissions throughout the 2017–2018 season and, unusually, all four subtyped viruses (A/H1, A/H3, and both B lineage viruses) were identified in meaningful numbers.

Our study and others [5] have found that very few patients hospitalized for ILI had received their seasonal influenza vaccination. However, because our study included only hospitalized cases of influenza, this does not reflect the national vaccination coverage rate. Nevertheless, Romania’s overall vaccine coverage remains very low among most risk groups [8–10]. Five patients with laboratory-confirmed influenza had received the 2017–2018 influenza vaccine 14 days or longer before symptom onset, and all five of these patients had received a trivalent vaccine. The dominant B/Yamagata lineage, which was not included in the 2017–2018 trivalent vaccine [18], was detected in four of these cases. Although the 2017–2018 formulation was estimated to have 36%–54% effectiveness against circulating B viruses (suggesting some cross-protection against the B/Yamagata lineage) [18], it is conceivable that these cases could have been avoided had the vaccine matched the circulating B lineage. The co-circulation of B/Victoria and B/Yamagata lineages has complicated the selection of the B strain to be included in trivalent influenza vaccines ahead of each season, and this has resulted in frequent global mismatches between the vaccine and the predominant circulating B strain [1, 20, 21]. Hence, our findings support the use of quadrivalent influenza vaccines, which include both B lineages, to help prevent mismatches in future seasons in Romania.

Our study provides important data on hospitalizations and severe illness associated with influenza in Romania, and helps address the lack in epidemiologic influenza data in this country identified by others [4, 5]. Additionally, our study reports the lineage of circulating influenza B viruses, which so far has not been reported in Romania’s national surveillance data from each season [17]. However, because our study collected data only from patients hospitalized with ILI, the results are limited to severe influenza cases. In addition, the data in this study were collected at a single hospital and only from inhabitants living within its catchment area. Although these factors could have caused differences from other regions of the country, the study area includes ~ 10% of the country’s population, and the country’s densely populated capital city, Bucharest [22]. Moreover, the influenza virus circulation patterns in our study were consistent with those reported for Romania as a whole [17, 18]. Finally, the A strain or B lineage were not identified in all cases and the neuraminidase for A virus strains was not determined, which could limit interpretation of influenza virus distribution.

## Conclusions

Influenza accounts for a considerable proportion of hospital admissions for ILI in Romania’s densely populated Bucharest-Ilfov region. Most patients hospitalized with influenza were young children or adults with comorbidities, and associated healthcare use was substantial. These findings suggest that measures should be taken to improve vaccine coverage in Romania, especially for children younger than 5 years. The data from this study should build on the limited epidemiologic information regarding Romania’s influenza burden and could be used to inform and optimize national influenza healthcare policies.

## Supplementary information

**Additional file 1: Table S1.** Characteristics of influenza-positive vs. influenza-negative patients.

## Data Availability

The datasets generated and analyzed during the study are available from the corresponding author on reasonable request.

## Abbreviations

ICD-10: International Classification of Diseases 10th revision
ICU: Intensive care unit
ILI: Influenza-like illness
IQR: Interquartile range
RSV: Respiratory syncytial virus
