# Supplementary material for: The 2017–2018 influenza season in Bucharest, Romania: epidemiology and characteristics of hospital admissions for influenza-like illness
Source: BMC Infect Dis. 2019 Nov 12;19:967. doi: 10.1186/s12879-019-4613-z (PMC6852761; doi:10.1186/s12879-019-4613-z)
Supplement: Supplementary file 1 — Additional file 1: Table S1. Characteristics of influenza-positive vs. influenza-negative patients. [file 12879_2019_4613_MOESM1_ESM.pdf]

Additional file for Drăgănescu et al: *The 2017−2018 influenza season in Bucharest, Romania: epidemiology and characteristics of hospital admissions for influenza-like illness*

Supplemental Table 1. Characteristics of influenza-positive vs. influenza-negative patients

Characteristic	Influenza-positive N=259	Influenza-negative N=243
Age (y), median [IQR]	6 [2.5−36]	5 [2−35.5]
Age group, n (%)		
<1 y	19 (7.3)	20 (8.2)
1−2 y	46 (17.8)	65 (26.7)
3−4 y	42 (16.2)	35 (14.4)
5−13 y	66 (25.5)	37 (15.2)
14−17 y	5 (1.9)	2 (0.8)
18−64 y	66 (25.5)	69 (28.4)
≥65 y	15 (5.8)	15 (6.2)
Sex, n (%)		
Male	133 (51.4)	111 (45.7)
Female	126 (48.6)	132 (54.3)
Smoking status, n (%)[a]		
Never smoked	143 (55.2)	139 (57.2)
Past smoker	45 (17.4)	35 (14.4)
Current smoker	71 (27.4)	69 (28.4)
Comorbidities, n (%)		
Any	50 (19.3)	45 (18.5)
Cardiovascular disease	28 (10.8)	26 (10.7)
COPD	6 (2.3)	4 (1.6)
Asthma	7 (2.7)	3 (1.2)
Diabetes	9 (3.5)	10 (4.1)
Immunodeficiency	5 (1.9)	13 (5.3)
Renal impairment	5 (1.9)	4 (1.6)
Autoimmune disease	7 (2.7)	5 (2.1)
Neuromuscular disease	6 (2.3)	8 (3.2)
Cirrhosis/liver disease	9 (3.5)	13 (5.3)
Neoplasm	3 (1.2)	3 (1.2)
Pregnant, n (%)	3 (1.2)	4 (1.6)
ILI symptoms (≥5-year-olds only), n (%)[b]		
Fever	136 (89.5)	106 (86.2)
Malaise	137 (90.1)	115 (93.5)
Headache	110 (72.4)	92 (74.8)
Myalgia	87 (57.2)	75 (61.0)
Cough	141 (92.8)	95 (77.2)
Sore throat	96 (63.2)	76 (61.8)
Shortness of breath	55 (36.2)	50 (40.7)
Polypnea (0−4-year-olds only), n (%)[c]	9 (8.4)	19 (15.8)
Hospitalizations in previous 12 mo, n (%)		
1	54 (20.8)	50 (20.7)[d]
≥2	33 (12.7)	35 (14.5)[d]
Outpatient consultations in previous 3 mo, n (%)		
1	56 (21.7)[e]	45 (18.7)[f]
≥2	71 (27.5)[e]	85 (35.3)[f]
Vaccinated against influenza in 2017−2018, n (%)	10 (3.9)	7 (2.9)
≥14 days before ILI onset	5 (1.9)	6 (2.5)

Duration of hospitalization (days), median [IQR]	5 [3−6]	5 [3.8−7][g]
Antiviral treatment prescribed, n (%)	246 (95.0)	208 (85.6)
Intensive care received, n (%)		
ICU admission	8 (3.1)	3 (1.2)
Mechanical ventilation	1 (0.4)	0 (0.0)
Death while hospitalized, n (%)	1 (0.4)	0 (0.0)

Abbreviations: COPD, chronic obstructive pulmonary disease; ICU, intensive care unit; ILI, influenza-like illness; IQR, interquartile range

[a] For patients aged 0−13 years of age, smoking habits refer to parents or tutors

[b] Percentage of patients aged ≥5 years (N=152 for influenza-positive, N=123 for influenza-negative)

[c] Percentage of patients aged 0−4 years (N=107 for influenza-positive, N=120 for influenza-negative)

[d] N=242

[e] N=258

[f] N=241

[g] N=240
